# Real-Time Arabic Sign Language Recognition Using a Hybrid Deep Learning Model

**DOI:** 10.3390/s24113683

**Published:** 2024-06-06

**Authors:** Talal H. Noor, Ayman Noor, Ahmed F. Alharbi, Ahmed Faisal, Rakan Alrashidi, Ahmed S. Alsaedi, Ghada Alharbi, Tawfeeq Alsanoosy, Abdullah Alsaeedi

**Affiliations:** Department of Computer Science, College of Computer Science and Engineering, Taibah University, Madinah 42353, Saudi Arabia; anoor@taibahu.edu.sa (A.N.); tu4101709@taibahu.edu.sa (A.F.A.); tu4107228@taibahu.edu.sa (A.F.); tu4102973@taibahu.edu.sa (R.A.); tu4101162@taibahu.edu.sa (A.S.A.); gfharbi@taibahu.edu.sa (G.A.); tsanoosy@taibahu.edu.sa (T.A.); aasaeedi@taibahu.edu.sa (A.A.)

**Keywords:** deep learning, real-time detection, Arabic sign language recognition, CNNs, LSTM

## Abstract

Sign language is an essential means of communication for individuals with hearing disabilities. However, there is a significant shortage of sign language interpreters in some languages, especially in Saudi Arabia. This shortage results in a large proportion of the hearing-impaired population being deprived of services, especially in public places. This paper aims to address this gap in accessibility by leveraging technology to develop systems capable of recognizing Arabic Sign Language (ArSL) using deep learning techniques. In this paper, we propose a hybrid model to capture the spatio-temporal aspects of sign language (i.e., letters and words). The hybrid model consists of a Convolutional Neural Network (CNN) classifier to extract spatial features from sign language data and a Long Short-Term Memory (LSTM) classifier to extract spatial and temporal characteristics to handle sequential data (i.e., hand movements). To demonstrate the feasibility of our proposed hybrid model, we created a dataset of 20 different words, resulting in 4000 images for ArSL: 10 static gesture words and 500 videos for 10 dynamic gesture words. Our proposed hybrid model demonstrates promising performance, with the CNN and LSTM classifiers achieving accuracy rates of 94.40% and 82.70%, respectively. These results indicate that our approach can significantly enhance communication accessibility for the hearing-impaired community in Saudi Arabia. Thus, this paper represents a major step toward promoting inclusivity and improving the quality of life for the hearing impaired.

## 1. Introduction

Sign language is a communication method utilized by deaf individuals and encompasses a series of hand gestures and symbols [1]. It is also employed by hearing individuals to facilitate communication with the deaf community. Predominantly, sign language is concept-based, with each gesture or symbol representing a distinct idea or concept. It uses four major manual components that comprise (1) finger configuration, (2) hand movement, (3) hand orientation, and (4) hand location relative to the body [2,3]. Compared to other gestures, sign language is the most structured. On the one hand, it has a large set of signs where each sign has a specific meaning [4]. On the other hand, this contrasts with word-based communication systems. Nonetheless, certain words and names lack direct equivalents in sign language. To address this, the deaf community often resorts to using a hand (i.e., finger) alphabet to spell out such words, ensuring clarity and precision in communication. This approach highlights the adaptability and inclusivity of sign language as a communication tool [5].

The Kingdom of Saudi Arabia is home to a sizable deaf population of about 229,541, many of whom are not provided with appropriate care in public venues because of a lack of interpreters, as the General Authority for Statistics has shown in recent years [6]. According to the Center for Strategic and International Studies (CSIS) [7], in the state of California, the ratio of sign language interpreters to hearing-impaired individuals is approximately 1:46. This indicates a relatively high availability of interpreters for the deaf community. In contrast, Saudi Arabia presents a starkly different scenario, with the ratio being approximately 1:93,000. This vast disparity highlights significant differences in the availability of sign language interpretation services between the two regions, underscoring a potential area of concern in terms of accessibility and support for the hearing-impaired population in Saudi Arabia. Moreover, most current research utilizes either only letters/alphabets to translate Arabic Sign Language (ArSL) or sensors to facilitate this process [4,8,9]. Thus, there is a need to conduct further research on ArSL [9,10].

In addressing the shortage of sign language interpreters, the role of technology, particularly machine-based communication methods, becomes crucial in bridging this gap. Advances in machine learning have led to the development of automated sign language translation systems. These systems employ sophisticated algorithms and gesture recognition technologies to translate sign language into spoken and written language, and vice versa. Such technological solutions offer a promising path to alleviate interpreter scarcity, particularly in areas such as the Kingdom of Saudi Arabia, where the ratio of interpreters to hearing-impaired individuals is exceedingly low. Machine-based communication can provide real-time, on-demand translation services, making communication more accessible and inclusive for the deaf community.

Furthermore, contemporary technological approaches, such as Convolutional Neural Networks (CNNs) and Long Short-Term Memory (LSTM), are instrumental in enhancing the efficacy of automated ASL translation systems. CNNs can process and interpret visual information, making them highly suitable for recognizing and analyzing the intricate hand gestures and facial expressions inherent in sign language [11,12,13,14]. LSTM networks, a form of recurrent neural network, excel in handling sequential data, thus effectively capturing the dynamic and temporal aspects of sign language [13,14,15,16]. Hence, in this paper, both CNNs and LSTMs were employed to develop the proposed model.

The novelty and main contribution of this paper focus on tackling a critical societal issue: the lack of sign language interpreters in the Kingdom of Saudi Arabia, which has left a significant hearing-impaired population without adequate support in public spaces. This research highlights the importance and versatility of Arabic Sign Language (ArSL) as a communication tool and leverages technology such as artificial intelligence to improve accessibility for the deaf community in Saudi Arabia. The key contributions of this paper include:

- Identifying the Accessibility Gap: This research comprehensively analyzes the accessibility gap faced by the hearing-impaired population in Saudi Arabia compared to regions with more interpreters such as California, USA. By highlighting the stark contrast in interpreter ratios, this paper sheds light on a crucial issue that requires attention and action.
- Leveraging Deep Learning: We propose a novel hybrid model to capture the spatio-temporal aspects of sign language. The hybrid model consists of a CNN classifier to extract spatial features from sign language data and an LSTM classifier to extract spatial and temporal characteristics to handle sequential data. This hybrid model facilitates the process of automatic sign language recognition in real time from either spoken or written language, addressing the shortage of qualified interpreters.
- Building a Custom Annotated Dataset: We create a dataset of 20 different universal words, resulting in 4000 images for ArSL. It consists of 10 static gesture words and 500 videos for 10 dynamic gesture words. The dataset can be used to train and evaluate ASL recognition models.
- Validation: We conduct a comprehensive evaluation of the hybrid model on our dataset and compare its performance on 20 different words (i.e., 10 static gesture words and 10 dynamic gesture words).

The remainder of this paper is organized as follows. The related works are covered in Section 2. Section 3 reviews the system architecture and the hybrid model. Section 4 describes the proposed model’s implementation, and Section 5 discusses the experimental results. Section 6 presents the concluding remarks and discusses future work.

## 2. Related Works

The issue of sign language recognition has recently attracted the attention of many researchers. Several survey articles have been specifically written to draw attention to the problems in the field of sign language recognition [1,10,17,18,19]. Some researchers have focused on ArSL recognition. For example, in [20], the researchers developed a real-time system for ArSL recognition using You Only Look Once (YOLO), in particular, the YOLOv5 model. The process began with a dataset of 5600 images of 28 ArSL signs, which was expanded to 15,088 images through augmentation techniques, including resizing, normalization, Gaussian blur, noise addition, affine transformations, and grayscale conversion. Three versions of the YOLOv5 model (small, medium, and large) were trained and evaluated, with the YOLOv5-large version achieving the best performance, with 99.5% in precision, 99.4% in recall, and 99.4% in mean average precision (mAP@.5). The YOLOv5s model, however, was identified as the most suitable for real-time recognition due to its high speed, with an average of 121 FPS and satisfactory mAP results.

In [21], the authors developed an Alphabet Recognition System for ArSL using a faster Region-based Convolutional Neural Network (RCNN). They collected a non-depth camera dataset of 15,360 images, containing hand movements against different backgrounds, captured using standard phone cameras to evaluate the system. The data were divided in such a way that 60% was used for training. Meanwhile, 20% of the total image collection was allocated for 3-fold cross-validation. The remaining 20% was set aside for testing. The proposed approach achieved 93% accuracy.

In [22], the authors developed a recognition system for Arabic, specifically Egyptian Sign Language (ESL), to improve communication with people with hearing impairment. It was implemented in a video-based system to serve the local deaf community in Egypt, utilizing two different neural network architectures: the first was a Convolutional Neural Network (CNN) to extract spatial features, and the second consisted of a CNN followed by Long Short-Term Memory (LSTM) to extract spatio-temporal features. The authors created a dataset by capturing videos under different lighting conditions, angles, and camera positions. The dataset consisted of nine classes, with videos recorded by two signers at five different locations. Each location contributed 20 videos, resulting in a total of 100 videos per chapter. Thus, the entire dataset included 900 videos. The researchers evaluated the performance of the two architectures. Using only the CNN, they achieved up to 90% accuracy. However, when they combined the CNN with LSTM, the accuracy dropped to 72%.

In [23], the authors developed a system to recognize ArSL letters. They used a CNN classifier to extract the exact position of the hand. The authors used an external dataset containing 1160 images of 416 × 416 pixels and merged it with a dataset they created containing 28 classes, where each category contained about 135 images describing an Arabic letter. The dataset was split, with 60% of the data used for training, 20% for testing, and 20% for validation. Their system achieved an accuracy of 97.07%.

Unlike previous research studies that focus solely on either image-based or video-based ArSL recognition, in this paper, we propose a hybrid model that can recognize ArSL in both forms. Furthermore, unlike previous research works that train their models on the 28 Arabic alphabet letters or only train their models on 9 Arabic words, our hybrid model is trained on 20 different Arabic words for videos and images using our dataset containing 4000 images and 500 videos.

Some researchers have focused on the recognition of other sign languages, such as English, Indian, Japanese, Bangla, Indonesian, Korean, Indian (Assamese), and Turkish. For example, [24] focuses on American-English Sign Language Translation (ASLAT), where various datasets and YOLO network models were utilized to enhance the accuracy and efficiency of sign language recognition. The open-access ASL alphabet dataset was partially used, with 24,000 images out of a total of 87,000, covering 26 ASL alphabet letters, excluding “J” and “Z”, and included additional signs for SPACE, DELETE, and NOTHING, though specific accuracy figures for this dataset were not mentioned. Another key component was the ASLYset dataset, comprising 5200 images and featuring 24 ASL alphabet signs (also excluding “J” and “Z”) and two extra signs, “SP” and “FN”. The testing dataset contained 1300 images with 50 images per sign, excluding “J” and “Z”. In terms of YOLO network models, two architectures, namely A1 and A2, were tested (A1 based on YOLOv3-tiny and A2 based on YOLOv3) with input image sizes of 288 × 288, 352 × 352, and 416 × 416 pixels. The highest mAP achieved by one of the YOLO models was 81.76%, although specific accuracy details for each model were not provided. The Bidirectional LSTM for Spelling Correction trained on a dataset based on 235 English sentences and expanded to 11,750 training sentences through noisy word generation showed a training accuracy of 98.07%.

In [25], the research aimed at classifying Indian Sign Language gestures, and an image database was created with images of hands of varying sizes and complexions. These images, originally sized at 768 × 1024, were pre-processed and resized to 256 × 256 to facilitate dimensionality reduction, which is crucial for faster classification. Principal Component Analysis (PCA) was employed for this purpose, not only reducing the time and storage space required but also improving the performance of the machine learning model by removing multi-collinearity and making the data easier to visualize. The study utilized two machine learning techniques to train the system: the K-Nearest Neighbor (KNN) algorithm and the backpropagation algorithm. The dataset comprised 220 images of double-handed and 800 images of single-handed Indian Sign Language alphabets, representing English letters and numbers. Using the KNN technique with K = 1, a 100% pattern recognition rate was accomplished, whereas the backpropagation technique yielded a recognition rate between 94 and 96%.

In [26], a sophisticated system for Japanese Sign Language (JSL) recognition was introduced, leveraging the Microsoft Kinect v2 sensor to capture sign motion feature parameters. The system utilizes a JSL dictionary, which encompasses a comprehensive vocabulary of approximately 2600 signs to categorize a variety of hand poses. An integral component of the system is its adoption of a contour-based technique for hand pose recognition, innovated by Keogh, which is particularly adept at handling situations where fingers are partially occluded. The experimental phase of the research involved the analysis of 892 samples, derived from 223 distinct signs, each performed by two interpreters and repeated twice, although the number of images was unspecified. These samples were instrumental in capturing the nuances of sign motion and hand positions. The findings from these experiments highlighted an improvement in recognition rates, with an increase in the number of wedges utilized. Despite a recognition accuracy of 33.8% for signs not previously included in the training data, the paper did not detail the dataset sizes for the employed models, such as Hidden Markov Models (HMMs) and Gaussian Mixture Models (GMMs), which were used for recognizing hand movement and position. This research marks a significant step forward in the domain of sign language recognition by presenting a real-time system that seamlessly integrates the elements of motion, position, and pose for JSL recognition.

In [27], the authors proposed a hand gesture recognition system, where a webcam was employed to collect a dataset comprising 2000 images of American-English Sign Language (ASL) gestures, specifically focusing on 10 static alphabet signs (A, B, C, D, K, N, O, T, Y), captured under various lighting conditions to ensure robustness. The system leverages advanced image processing techniques, including HSV color extraction for effective background removal, alongside segmentation and morphological operations to refine the quality of the images. These pre-processed images are then fed into a Convolutional Neural Network (CNN), which is trained on 1600 of these images, with the remaining 400 images reserved for testing, adhering to an 80:20 training-to-testing ratio. The result is a highly accurate gesture recognition system, evidenced by a remarkable 100% prediction accuracy rate achieved by the CNN model, showcasing its capability to accurately identify gestures from images. This system not only demonstrates the potential of CNNs in image-based recognition tasks but also highlights the importance of comprehensive data collection and meticulous pre-processing in developing effective recognition algorithms. The dataset comprised only 2000 photos, averaging 200 images for each gesture.

In [28], the researchers analyzed and interpreted Indian Sign Language using LSTM networks, employing a dataset representing 40 different actions, with 30 videos for each action, each containing 30 frames, ensuring diversity in gender and signing capabilities. The videos were recorded using high-resolution cameras in controlled environments to guarantee the quality and realistic representation of the data. The data underwent multiple stages, including video processing and feature extraction, utilizing techniques such as Principal Component Analysis (PCA) and wavelet analysis, paving the way for the application of LSTM networks to learn the temporal patterns of the signs. Despite challenges and limitations related to the dataset’s size and the full representation of sign language diversity, the study achieved a training accuracy of 87%, indicating the model’s ability to accurately classify the signs. In [29], the authors proposed an approach to train and detect Bengali Sign Language using deep learning. The authors used a CNN classifier to train each sign individually. The system was trained on samples containing different signs used in Bengali Sign Language. They captured their dataset using an Intel RealSense webcam. They labeled 90 classes in total, and each class represented one label. The data were split into 80% for training and the remaining 20% for testing and validation. The accuracy of their model reached 78%.

In [30], the authors proposed a YOLOv3 method for Indonesian Sign Language recognition. The authors developed a system that can automatically translate sign language into text to facilitate communication between deaf individuals and those who do not understand sign language. The system was designed to process video data inputs in real time using an object detection method, such as YOLOv3, based on a CNN classifier. The dataset used in this research was collected and captured independently by the authors under different conditions and based on Indonesian Sign Language, and it consisted of images and videos representing 24 categories of sign language gestures. The dataset contained approximately 160 to 220 pictures, representing the letters A to Z, excluding J and R. When using image data, the data were split into 80% for training and 20% for testing. Their system achieved 100% accuracy. When using video data, the system achieved 72.97% accuracy.

In [31], the authors developed a video data input processing system for Korean Sign Language recognition by using human key points extracted from the face, hand, and body parts as input for a recurrent neural network (RNN). They presented a dataset consisting of 10,480 high-resolution, high-quality video clips. They trained and tested their model using a dataset of 1000 videos, each recording 100 different sign language sentences. The system achieved a classification accuracy of 89.5% for 100 sentences that could be used in emergencies. This suggests that the system can be particularly useful in critical situations where clear communication is essential.

To overcome the difficulties in sign language recognition, the authors of [32] proposed an end-to-end skeleton-based multi-feature multi-stream multi-level information sharing network (TMS-Net). Combining joint information with global features, bone features with local features, and angle features with scale invariance allows TMS-Net to improve input richness. Comprehensive extraction and exploitation of skeleton feature information are ensured by its multi-stream structure and multi-level information-sharing mechanism. Based on a single modality input, the experimental results demonstrated that TMS-Net outperformed state-of-the-art approaches on the WLASL-2000 (56.4%), AUTSL (96.62%), and MSASL (65.13%) datasets. Furthermore, the practical efficiency of TMS-Net was demonstrated in an SLR-based Human–Robot Interaction (HRI) experiment, underscoring its potential for real-world applications.

In [33], the authors proposed the Natural Language-Assisted Sign Language Recognition (NLA-SLR) framework as a solution to the issue of visually indistinguishable signs (VISigns) in sign languages. They introduced language-aware label smoothing, which generates soft labels with smoothing weights based on normalized semantic similarities among glosses, to improve training for VISigns with similar semantic meanings. They provided an inter-modality mix-up approach that combines vision and gloss data for VISigns with different semantic meanings, improving separability under blended label supervision. Moreover, a unique backbone, the video-keypoint network, extracts knowledge from sign videos across various temporal receptive fields by modeling both RGB films and human body key points. The empirical findings revealed that NLA-SLR is effective in enhancing the accuracy of sign language recognition, achieving state-of-the-art performance on the MSASL, WLASL-2000, and NMFs-CSL datasets, achieving an average accuracy of 61.26% on the WLASL-2000 dataset. Unlike previous research that proposed NLA-SLR, achieving 61.26% accuracy, this study offers a hybrid approach for Arabic Sign Language (ArSL), achieving an average accuracy of 88.55% for both sub-models.

In [34], the authors presented two deep neural network-based model designs to address the challenges in gesture recognition and Audio-Visual Speech Recognition (AVSR). The main innovations in AVSR are the end-to-end model that utilizes three modality fusion approaches—prediction-level, feature-level, and model-level—as well as the methodologies for fine-tuning both visual and audio features. The deep neural network-based model consists of two architectures: (i) a visual model architecture that leverages Two-Dimensional Convolutional Neural Networks and Bidirectional Long Short-Term Memory (2DCNN+BiLSTM), Three-Dimensional Convolutional Neural Networks (3DCNN), or Three-Dimensional Convolutional Neural Networks and Bidirectional Long Short-Term Memory (3DCNN+BiLSTM), and (ii) an audio model architecture that leverages Residual Networks (ResNets), Visual Geometry Group (VGG), or Pretrained Audio Neural Networks (PANNs). The novel aspect of gesture identification lies in a distinct set of spatio-temporal features (STF), which take lip articulation information into account. The experimental results showed the performance of the models on two large-scale corpora, obtaining an AVSR accuracy of 98.76% on the LRW dataset and a gesture recognition rate of 98.56% on the AUTSL dataset, despite the lack of combined task datasets. Unlike previous research that focused on Audio-Visual Speech Recognition (AVSR), this study focuses on Arabic Sign Language (ArSL).

In [35], the authors focused on the issue of conventional machine learning techniques requiring significant data collection and classification. They presented a novel deep learning architecture, namely 3D-CLDNN, that makes use of sEMG signals and depth vision. By automatically classifying depth data using a self-organizing map and predicting gestures using only sEMG data, it simplifies the procedure. With its 84.4% accuracy rate and fast processing time, the approach is appropriate for real-time applications involving human–machine interaction.

In [36], the authors developed a method for recognizing Indian (Assamese) Sign Language. They prepared a dataset of 2D and 3D images of Assamese gestures containing nine Assamese alphabets captured using a Microsoft Kinect sensor and an RGB webcam. They used the MediaPipe framework to detect landmarks in images and trained them using a feedforward neural network. Their model achieved up to 99% accuracy. However, there was no mention of the size of the dataset used. Unlike previous research works that focus on the recognition of other sign languages such as English, Indian, Japanese, Bangla, etc., in this paper, we focus on the recognition of Arabic Sign Language (ArSL). Moreover, unlike previous research works that focused on either image-based or video-based sign language recognition, in this paper, we propose a hybrid model that can recognize ArSL in both forms.

In [37], the authors addressed the issue of clearly delineating boundaries for sign words in systems for Continuous Sign Language Recognition (CSLR) and Sign Language Translation (SLT). They observed that traditional methods based on pre-trained models are less flexible and computationally demanding, but intermediate gloss prediction greatly improves SLT performance. In response to these problems, they suggested the Sign2Pose gloss prediction transformer, which lowers processing overhead and increases accuracy by using manually developed posture feature extraction techniques. To effectively detect key frames in sign movies, the authors used the Euclidean distance technique and a modified version of the HD algorithm. By including YOLOv3 to accurately detect hand movements, their model outperformed current pose-based techniques by 15–20% in accuracy. Their model achieved a new standard for accuracy and efficiency by outperforming other methods on the word-level ASL data corpus. Unlike previous research that proposed the Sign2Pose gloss prediction transformer model, which scored 80.90% in accuracy, this study offers a hybrid approach for Arabic Sign Language (ArSL), achieving an average accuracy of 88.55% for both sub-models.

In [38], the authors concentrated on the issue of large vocabulary in practical contexts for recognizing sign language. The authors presented a large-scale multi-modal Turkish Sign Language dataset, namely Ankara University Turkish Sign Language (AUTSL), which included baseline and benchmark models for evaluating performance. The dataset consisted of 226 signs executed by 43 signers, for a total of 38,336 isolated sign video clips. Microsoft Kinect v2 was used to record the RGB, depth, and skeleton modalities for each sample. The authors trained multiple deep learning models using CNNs for feature extraction and both unidirectional and bidirectional LSTMs to capture temporal information, which was then improved by feature-pooling modules and temporal attention. They also created benchmark training and testing sets for user-independent evaluations. The reported results on the AUTSL and Montalbano datasets were competitive, with accuracies of 96.11% and 95.95%, respectively. The limitations inherent in the user-independent benchmark dataset were highlighted, with the best baseline model achieving an accuracy of 62.02%. The authors focused on recognizing Turkish Sign Language, whereas our paper concentrates on the recognition of Arabic Sign Language (ArSL).

## 3. System Architecture and the Hybrid Model

**Figure 1:**
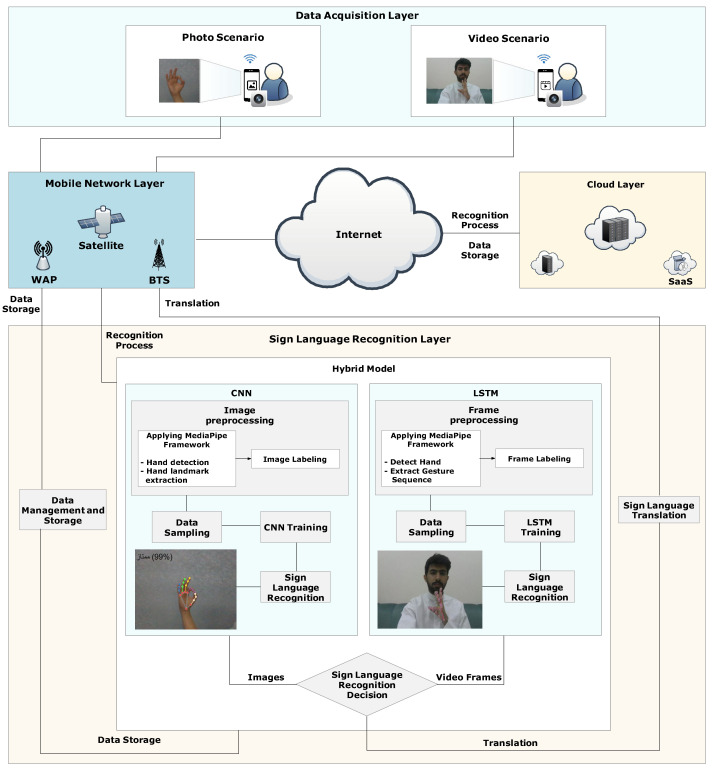
Real-time ArSL system architecture.

To overcome the lack of Arabic interpreters for people with hearing impairment, especially in Saudi Arabia, an architecture for sign language recognition is presented in this work that enables automatic real-time translation between sign language and spoken or written language. The architecture is shown in Figure 1 and consists of four layers: the data acquisition layer, the mobile network layer, the cloud layer, and the sign language recognition layer.

### 3.1. Architecture Layers

Each layer of the architecture is responsible for a set of tasks and interacts with the other layers. The architecture uses a streamlined pipeline design to make communication accessible and intuitive. The architecture layers are as follows:

*1. Data Acquisition Layer:* This layer gathers the sign language data that require translation using images collected from cameras (e.g., webcams, smartphone cameras, wearable gadget cameras, etc.) or videos collected from cameras, which are broken into frames. The images and frames are then sent through the *mobile network layer* and the *sign language recognition layer* for processing.

*2. Mobile Network Layer:* This layer connects the *data acquisition layer* and the *sign language recognition layer*. It is made up of several Wireless Access Points (WAPs), Base Transceiver Stations (BTSs), and satellites to enable communication. The information that is provided includes the ID of the camera and the images or video frames that contain the hand landmark or gesture sequence that needs to be translated. This layer makes communication easy and straightforward through a streamlined pipeline design.

*3. Cloud Layer:* For the other layers, this layer offers Infrastructure as a Service (IaaS) and Software as a Service (SaaS), making it possible for data to be stored and shared across layers via the Internet. It also gives the system security, scalability, and dependability. By employing a hybrid model for processing the deep learning models, including the training and learning phases, we use SaaS, a cloud computing model that does not require management of the underlying infrastructure. The management and storage of data, including training and recognition data for upcoming training, are achieved using the IaaS cloud computing architecture. With this computing approach, resources like servers, storage, and networks can grow as needed without needing to be managed.

*4. Sign Language Recognition Layer:* This layer utilizes the proposed *hybrid model* on each image or video frame that comes from the *data acquisition layer*, which contains a hand landmark or gesture sequence to be translated. The *hybrid model* consists of two deep learning models, namely Convolutional Neural Networks (CNNs) and Long Short-Term Memory (LSTM), each of which consists of several modules:

- **Image/Frame Pre-processing:** This module employs the Google MediaPipe framework (https://developers.google.com/mediapipe/framework, accessed on 23 February 2024) to extract hand landmarks or gesture sequences from a set of images or frames. The images or frames are organized into separate directories, each representing a unique category. For every image or frame, the coordinates of detected hand landmarks or gesture sequences are captured and flattened into a list. These lists are then compiled into a ’data’ list while corresponding category labels are added to the ’labels’ list. The entire dataset, consisting of hand landmarks or gesture sequences and their associated labels, is stored in a pickle file (i.e., in the *cloud layer*).
- **Data Sampling:** This module is a crucial aspect of deep learning, particularly when dealing with data that require training and testing for their models. It randomly splits the data into 80% for training and 20% for testing. This division ensures that both models (i.e., CNN and LSTM in the hybrid model) learn patterns and relationships from a diverse range of examples during the training phase while also assessing their performance on unseen data during testing. The training set, comprising 80% of the data, is used to train the models, which allows them to learn from a large variety of hand gestures and their corresponding landmarks. On the other hand, the remaining 20% of the data reserved for testing serves as an independent validation set to evaluate both models’ performance and generalization ability on new, unseen examples.
- **CNN Training:** The CNN sub-model of our *hybrid model* is designed to detect human hands by leveraging the Google MediaPipe framework to identify the hand’s 21 3D landmarks. These landmarks are crucial for understanding hand gestures and movements. To train the CNN sub-model, we utilize pre-processed data stored in a pickle file (i.e., in the *cloud layer*), where the hand landmarks and corresponding labels are organized. We then employ the Random Forest Classifier (RFC) to predict the output of these landmarks. The RFC is an ensemble learning algorithm that constructs multiple decision trees during training. It outputs the mode of the classes (i.e., classification) or the mean prediction (i.e., regression) of the individual trees. Each decision tree in the ensemble is trained on a random subset of the training data. During prediction, each tree contributes a decision, with the final output determined by a majority or averaging mechanism. This combined approach helps the CNN sub-model to accurately detect and interpret human hand gestures in real-time applications.
- **LSTM Training:** The LSTM sub-model of our *hybrid model* uses an approach that focuses on capturing sequential patterns in time-series data, particularly in the context of hand gesture sequences. To train the LSTM sub-model, we utilize pre-processed data stored in a pickle file (i.e., in the *cloud layer*), where the features and corresponding labels of the hand gesture sequences are organized. The features are then organized into sequences of frames, with each frame representing a window of historical data of the hand gesture sequence. We utilize the LSTM architecture, a type of recurrent neural network (RNN) designed to effectively model long-term dependencies in sequential data. During training, the LSTM sub-model learns to capture intricate patterns and relationships within the hand gesture sequences. We employ techniques such as dropout regularization to prevent overfitting and optimize the model’s generalization ability.
- **Sign Language Recognition:** This module is responsible for predicting the hand gestures based on the training information from both the CNN and LSTM models (i.e., *hybrid model*). Further details on the hybrid model are elaborated in Section 3.2.

### 3.2. Hybrid Model

Our proposed architecture is designed to gather the ArSL data that require translation using images or videos collected from cameras, displaying the corresponding text translation on screen in real time. We propose a hybrid model that consists of a CNN classifier to extract spatial features from ArSL data and an LSTM classifier to extract spatial and temporal characteristics to handle sequential data. To enable the user to use both images and videos, we created a function called “Sign Language Recognition Decision” to give our system the flexibility to use either images or videos. This function essentially chooses between the image processing sub-model (CNN) and the video processing sub-model (LSTM) depending on the input type. By integrating the strengths of CNNs and LSTMs, the system can leverage both spatial and temporal information simultaneously. The CNN extracts relevant visual features, and the LSTM processes these features in a sequential manner, capturing the dynamic aspects of sign language communication. This allows users to communicate naturally using gestures. More details on CNN and LSTM models are elaborated in the following subsection.

#### 3.2.1. Convolutional Neural Network (CNN)

**Figure 2:**
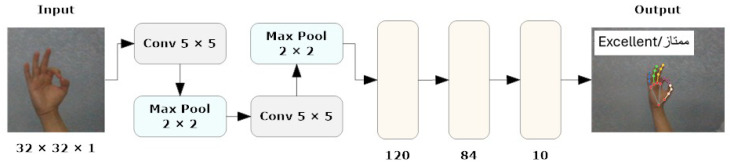
CNN model structure.

The CNN sub-model of our *hybrid model* is designed to detect human hands by leveraging the Google MediaPipe framework to identify the hand’s 21 3D landmarks. These landmarks are crucial for understanding hand gestures and movements. For the proposed CNN sub-model, we utilized LeNet-5, a well-known CNN architecture frequently employed for image recognition tasks. This architecture comprises several layers: it begins with two convolutional layers, each followed by max-pooling layers, and concludes with three fully connected (linear) layers. As shown in Figure 2, the input to LeNet-5 is a grayscale image with dimensions of 32 × 32 × 1, where the “1” denotes a single channel for grayscale intensity. The first convolutional layer applies six filters of size 5 × 5 to the input image, producing an output shape of [−1, 6, 28, 28]. The subsequent max-pooling layers halve the spatial dimensions, resulting in feature maps of sizes [−1, 6, 14, 14] and [−1, 16, 5, 5], respectively. The final three linear layers have output shapes of [−1, 120], [−1, 84], and [−1, 10], with the last layer representing the output classes. LeNet-5 consists of 61,706 parameters in total, including weights and biases, distributed across the convolutional and linear layers.

To train the CNN sub-model, we utilized pre-processed data stored in a pickle file. We then employed the Random Forest Classifier (RFC) to predict the output of these landmarks. Bootstrapping was used to sample a random subset of data and features used to train each tree in the forest. An aggregation of the predictions produced by every single decision tree results in the final RFC predictions. The final RFC prediction for hand gesture γ taking on sign language word class σ in the tree is explained in Equation (1):

(1) $$P_{\tau }\left[\sigma |\gamma ,\Omega ,\pi \right]=\sum_{\lambda \in \Lambda }\pi \lambda_{\sigma }\mu_{\lambda }\left(\gamma ,\Omega \right),$$

where Ω denotes the parameter of the decision function δ, π denotes the class label distribution of all leaf nodes, Λ denotes a set of leaf nodes, $\pi \lambda_{\sigma }\mu_{\lambda }$ denotes the probability that the hand gesture belongs to a sign language word class σ given by leaf node λ, and $\left(\gamma ,\Omega \right)$ denotes the probability of routing the hand gesture until leaf node λ. Ultimately, we can interpret this probability value as a weighted sum of the class distribution if we treat the possibility of reaching the leaf node as a weight. The decision function δ for split node ν is defined in Equations (2) and (3):

(2) $$\delta_{\nu }\left(\gamma ;\Omega \right)=\beta \left(f_{\nu }\left(\gamma ;\Omega \right)\right),$$

(3) $$\beta \left(\gamma \right)={\left(1+e^{-\gamma }\right)}^{-1},\;\;\;\;\;\;\;\;\;\;\;\;where\;\;\;\;\;\;f_{\nu }\left(.;\Omega \right)\;:\;\Gamma \to R$$

where $\beta \left(\gamma \right)$ denotes the sigmoid function, whose output can be used to calculate the possibility of moving to the left or right sub-tree in the RFC, and $f_{\nu }\left(.;\Omega \right)$ denotes the real-valued function parametrized by Ω.

The model’s ability to learn hand landmarks is encoded by the parameter Ω. The parameters of a deep CNN, which are used to automatically train an appropriate hand landmark representation from incoming images, are represented by Ω in this paper. Every function $f_{\nu }$ can be thought of as a deep network’s linear output unit. The final prediction of the CNN model, delivered by the forest $F=\{\tau_{1},\tau_{2},\ldots ,\tau_{\gamma }\}$ for the hand landmark γ, is calculated as shown in Equation (4):

(4) $$P_{F}\left[\sigma |\gamma \right]=\frac{1}{x}\sum_{n=1}^{X}P_{\tau_{n}}\left[\sigma |\gamma \right],$$

where γ denotes the hand gesture, σ denotes the sign language word class, *x* denotes the number of trees in the forest, and $P_{\tau_{n}}$ represents the probability that the hand gesture belongs to sign language word class σ given by the $n^{th}$ tree. The average of the prediction made by each tree in the forest eventually determines the final prediction.

#### 3.2.2. Long Short-Term Memory (LSTM)

**Figure 3:**
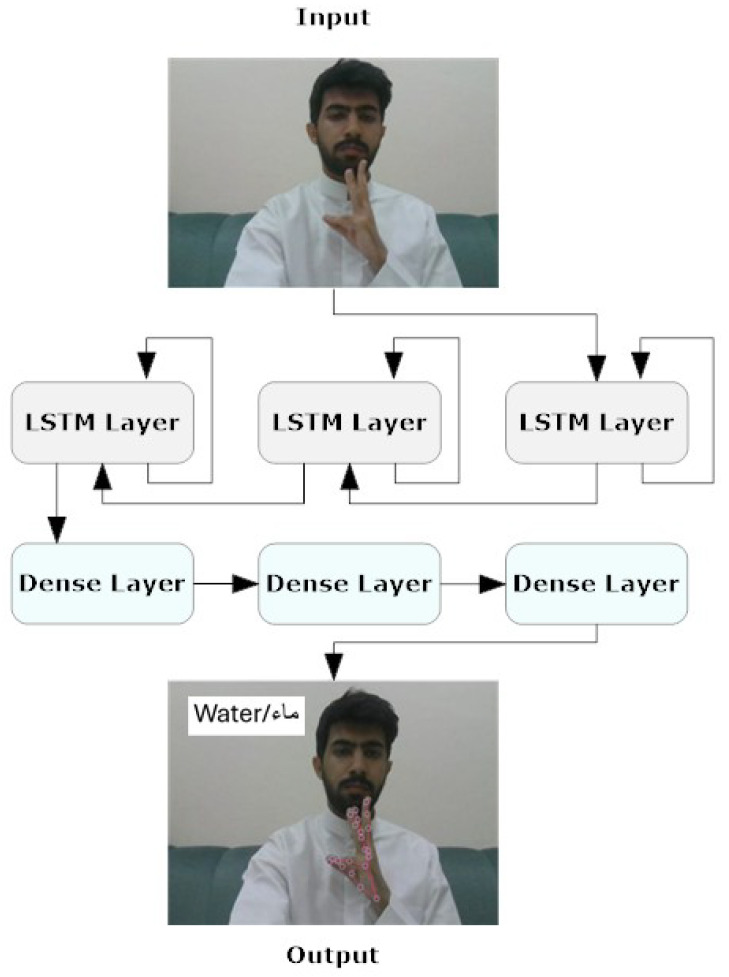
LSTM model structure.

LSTM is a type of recurrent neural network (RNN) designed to effectively model long-term dependencies in sequential data [39,40,41]. For the proposed LSTM sub-model, we employ a stack structure, which involves arranging multiple LSTM layers in sequence, where the output of each LSTM layer serves as the input for the next. This deep architecture allows for capturing more intricate patterns in sequential data by utilizing both LSTM and dense layers. As illustrated in Figure 3, the first LSTM layer has 64 units, returns sequences, and uses the hyperbolic tangent function (tanh) for activation. The second LSTM layer includes 128 units, returns sequences, and also uses tanh for activation. The third LSTM layer, with 64 units, does not return sequences and performs tanh activation. Following the LSTM layers are the dense layers: the first dense layer has 64 units and uses tanh activation, the second dense layer has 32 units with tanh activation, and the final dense layer has units equal to the number of actions, employing the Softmax function to constrain the outputs between 0 and 1, ensuring their sum is always 1.

During training, the LSTM sub-model learns to capture intricate patterns and relationships within the hand gesture sequences. In this paper, LSTM model is employed to learn hand gesture sequences and to recognize the correspondent sign language translation. leveraging the Google MediaPipe framework to extract input representations, the structure of LSTM is expressed in the following manner:

A form of RNN called LSTM architecture is intended to efficiently model long-term dependencies in sequential data [15,42,43]. During training, the LSTM sub-model picks up on the complex correlations and patterns found in the hand gesture sequences. To learn hand gesture sequences and recognize the corresponding sign language translation, this paper uses an LSTM model. Exploiting Google MediaPipe framework to extract input representations, the structure of LSTM consists of several gates including the input gate, the forget gate, the output gate, and the cell input vector. The input gate is described in Equation (5) as follows:

(5) $$\alpha_{t}=\psi \left(\omega_{\alpha }\cdot \left[h_{t-1},x_{t}\right]+b_{\alpha }\right),$$

where the input gate, denoted as α, is controlled by the time step, denoted as *t*. ψ denotes the logistic sigmoid function. The last hidden state, denoted as h[t−1], and the current input, denoted as $x_{t}$, are weighted using the matrix weight ω. $b_{\alpha }$ denotes the biases of the input gate. The forget gate is described in Equation (6), as follows:

(6) $$\varphi_{t}=\psi \left(\omega_{\varphi }\cdot \left[h_{t-1},x_{t}\right]+b_{\varphi }\right),$$

where the forget gate, denoted as φ, is controlled by the time step, denoted as *t*. Similar to the input gate, ψ denotes the logistic sigmoid function. The last hidden state, denoted as $h_{t-1}$, and the current input, denoted as $x_{t}$, are weighted using the matrix weight ω. $b_{\varphi }$ denotes the biases of the forget gate. As we can observe, the input gate’s mechanism is identical to this one, but it uses a completely different set of weights. The output gate is expressed in Equation (7), as follows:

(7) $$\beta_{t}=\psi \left(\omega_{\beta }\cdot \left[h_{t-1},x_{t}\right]+b_{\beta }\right),$$

where the output gate, denoted as β, is also controlled by the time step, denoted as *t*. Similar to the input and forget gates, ψ denotes the logistic sigmoid function. The last hidden state, denoted as $h_{t-1}$, and the current input, denoted as $x_{t}$, are weighted using the matrix weight ω. $b_{\varphi }$ denotes the biases of the output gate. The cell input vector is shown in Equations (8) and (9), as follows:

(8) $$\varepsilon_{t}=\varphi_{t}⊙\varepsilon_{t-1}+t_{i}⊙tanh\left(\omega_{\varepsilon }\cdot \left[h_{t-1},x_{t}\right]+b_{\varepsilon }\right),$$

(9) $$h_{t}=\beta_{t}⊙tanh\left(\varepsilon_{t}\right),$$

where tanh denotes the tangent function used to transform the data into a normalized encoding of the data. ⊙ represents the element-wise product of two vectors.

## 4. Implementation

For the implementation, we utilized Google Cloud Computing Services (Google Cloud) (https://cloud.google.com/, accessed on 28 February 2024). Within this environment, we deployed virtual machines running the Ubuntu Operating System version 20.04, sourced from the Ubuntu OS Cloud Marketplace (https://console.cloud.google.com/marketplace/product/ubuntu-os-cloud/ubuntu-focal, accessed on 28 February 2024). These virtual machines were configured with the Docker platform (https://cloud.google.com/compute/docs/containers, accessed on 28 February 2024) to execute the application container. In particular, we employed the standard c3d-Standard-4 configuration on Google Cloud, with each virtual machine featuring 4 VCPUs and 16 gigabytes (GB) of memory. Our system architecture consisted of three such virtual machines, each assigned distinct responsibilities for each layer in our architecture, including the data acquisition, sign language recognition, and cloud layers, as illustrated in Figure 1. Leveraging Google Cloud facilitated the setup and management of our system architecture, providing reliability, speed, and scalability. Additionally, Google Cloud offers a range of services and features tailored to our system architecture requirements and use cases. We also leveraged Google Cloud’s security and governance services to safeguard our data and applications against unauthorized access and potential threats. The configuration parameters and corresponding values chosen for setting up and managing our system architecture on Google Cloud are detailed in Table 1.

Upon initiating the c3d-Standard-4 instance, we proceeded to install Docker version 4.28.0 on the virtual machine (https://docs.docker.com/desktop/release-notes/#4280, accessed on 29 February 2024). Subsequently, we established three containers utilizing Arch Linux via Docker. Each container served a distinct purpose within our system architecture: the first container managed the data acquisition layer, the second container handled the sign language recognition layer, and the third container oversaw the cloud layer functionality. The data acquisition layer was responsible for gathering images or videos from various sources such as webcams, smartphone cameras, and wearable gadget cameras. The sign language recognition layer processed these data to interpret hand gestures. These containers were equipped with a Python interpreter version 3.12.2 (https://www.python.org/downloads/release/python-3122/, accessed on 1 March 2024) and NodeJS version 20.11.1 (https://nodejs.org/en/blog/release/v20.11.1, accessed on 1 March 2024), enabling us to develop and execute code for both the data acquisition and sign language recognition layers. Python facilitated the creation of concise and comprehensible code capable of handling intricate tasks and data structures. Meanwhile, NodeJS permitted the utilization of JavaScript for both front-end and back-end development, streamlining our codebase and enhancing performance. The final container utilized a Docker Compose file comprising an image for MySQL to effectively manage and store the data (https://hub.docker.com/_/mysql, accessed on 29 February 2024).

### 4.1. Dataset Description

In this paper, we employed a hybrid model that leverages deep learning techniques to achieve high performance and accuracy in gesture recognition tasks. To accomplish this, we utilized a large-scale dataset consisting of images and videos captured using built-in webcams with a resolution of 480p. For the CNN model, we collected 400 images for each of the 10 targeted words. Each image has dimensions of 640 × 480 pixels, ensuring a detailed representation of hand gestures. Additionally, for the LSTM model, we obtained 50 videos for each word (i.e., 10 words), totaling 500 videos. These videos, recorded with the same 480p webcam, provide dynamic visual sequences for temporal analysis. We used the most universal and commonly used words for sign language, like hello, water, teacher, work, etc. The translations of these words were taken from the Handspeak website (https://www.handspeak.com/, accessed on 12 February 2024) and the Saudi Sign Language website (https://sshi.sa/, accessed on 12 February 2024). The Handspeak website is an online resource for sign language, and the Saudi Sign Language website is a platform affiliated with the Saudi Society for Hearing Impairment. The diverse nature and scale of our dataset enabled robust training and evaluation of the hybrid model for accurate gesture recognition tasks.

### 4.2. Data Labeling and Model Training

We divided the dataset into 80% for training and 20% for testing to validate the proposed hybrid model.

#### 4.2.1. CNN Sub-Model

The dataset for training the CNN sub-model comprised images we captured, each depicting a specific hand gesture. We utilized MediaPipe to extract hand features from these images, focusing on 21 hand landmarks. These features were saved as .npy files in directories corresponding to each of the 10 distinct gesture labels in our dataset. Each label category contained 400 images, resulting in a balanced dataset of 4000 images. Of these, 3200 hand landmark images were manually labeled and categorized based on the selected 10 words (as shown in Table 2) to train the CNN sub-model of the proposed hybrid model. The remaining 800 hand landmark images were used to evaluate the CNN sub-model.

As detailed in Table 3, the CNN sub-model was trained using the following hyperparameters: a learning rate of 0.001 to ensure stable convergence, a batch size of 32 to balance memory usage and training speed, and a total of 50 epochs to allow for thorough training and convergence. We used the Adam optimizer, known for its efficiency and adaptive learning rates, and employed cross-entropy loss as the loss function, which is suitable for our classification task. To prevent overfitting and enhance the model’s generalization ability, we incorporated a dropout rate of 0.5. Dropout is a regularization technique that involves randomly ignoring selected neurons during training, which helps the model avoid relying too heavily on specific features or neurons and promotes robustness.

#### 4.2.2. LSTM Sub-Model

The dataset comprised recorded sequences of actions stored in directories corresponding to each specific action. It included 10 actions, each represented by 50 videos. Each sequence consisted of 30 frames, with each frame represented by a 1662-dimensional feature vector. These files were loaded into memory, and the sequences and their labels were compiled into arrays. We captured the videos in this dataset; 400 of the collected hand gesture sequence videos were manually labeled and categorized according to 10 selected words (as shown in Table 4) to train the LSTM sub-model of the proposed hybrid model, which was trained for 50 epochs. Additionally, 100 hand gesture sequence videos were used to test the LSTM sub-model.

Table 5 provides details of the LSTM sub-model’s training, including the parameters for the LSTM layers and dense layers. The first LSTM layer outputs a shape of (None, 30, 64) with 442,112 parameters, followed by a second LSTM layer with an output shape of (None, 30, 128) and 98,816 parameters. The third LSTM layer produces an output shape of (None, 64) with 49,408 parameters. This is followed by three dense layers: the first dense layer has an output shape of (None, 64) with 4,160 parameters, the second dense layer has an output shape of (None, 32) with 2,080 parameters, and the third dense layer has an output shape of (None, 10) with 330 parameters. In total, the model has 596,906 parameters.

## 5. Experimental Results

To demonstrate our proposed hybrid model’s applicability, we conducted several experiments to evaluate the performance of each sub-model of our proposed hybrid model, including the Convolutional Neural Network (CNN) and Long Short-Term Memory (LSTM) models.

### 5.1. Evaluation Metrics

In this section, we outline the evaluation criteria used to gauge the effectiveness of the hybrid model, which includes the Convolutional Neural Network (CNN) and Long Short-Term Memory (LSTM) models. Specifically, we examine important metrics such as accuracy, precision, recall, F1 score, confusion matrix, and loss. These metrics are essential for gaining a comprehensive understanding of the hybrid model’s predictive abilities and the sub-models’ ability to generalize. Alongside the traditional performance metrics, assessing loss provides insights into the optimization process and the convergence of the sub-models. It is worth emphasizing that all metrics were assessed using a distinct validation dataset, separate from the training data, to ensure the robustness and reliability of the hybrid model performance assessment.

### 5.2. CNN Evaluation Metrics

**Figure 4:**
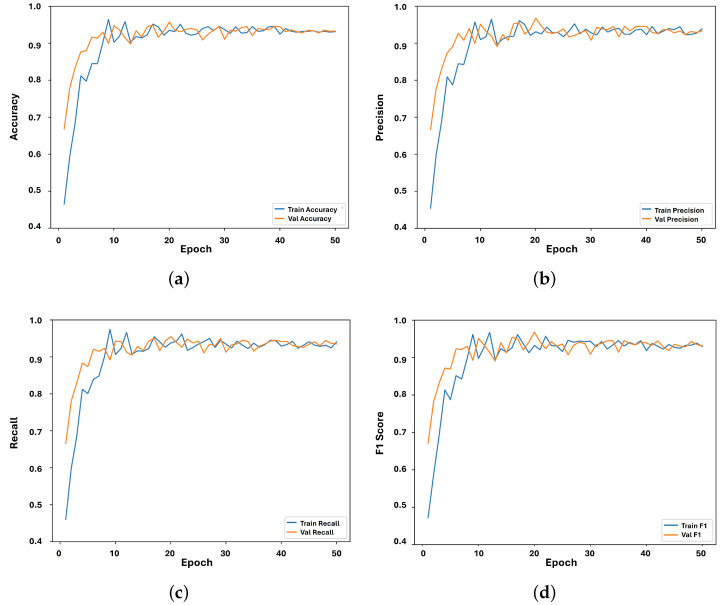
CNN sub-model performance evaluation. (**a**) CNN sub-model accuracy; (**b**) CNN sub-model precision; (**c**) CNN sub-model recall; (**d**) CNN sub-model F1 score.

Figure 4 illustrates the results of the performance of the CNN sub-model on the 10 words listed in Table 2. For this experiment, we trained the CNN sub-model using 3200 images (i.e., for hand landmark images) and validated it using 800 images. The accuracy of the CNN sub-model was 94.40% (see Figure 4a). The reason for this is the adequate amount of data for training, where the model could understand the hand gestures using the hand landmarks. The main purpose for setting the number of epochs to 50 was to leave room for the CNN sub-model to understand the targeted hand gestures. Additionally, the CNN sub-model achieved a precision of 95.00% (see Figure 4b) and recall and F1 scores of 94.40% and 94.90%, respectively, as shown in Figure 4c,d. The accuracy, precision, recall, and F1 scores consistently fell within the range of 91.00% to 95.00%. This uniformity in performance across these metrics indicates balanced model behavior. The similarity in accuracy, precision, recall, and F1 score can be attributed to the balanced nature of the dataset, where each class is adequately represented, facilitating equitable performance evaluation across all classes.

**Figure 5:**
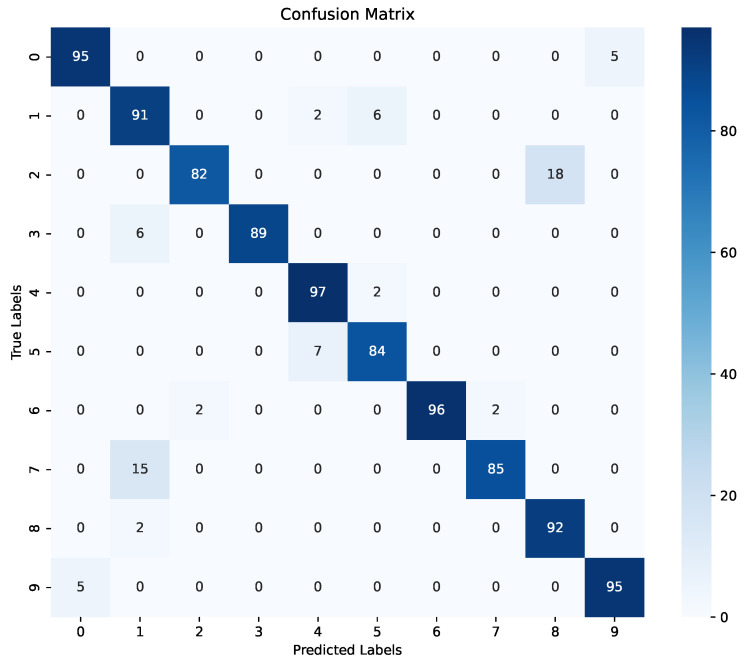
CNN sub-model confusion matrix.

**Figure 6:**
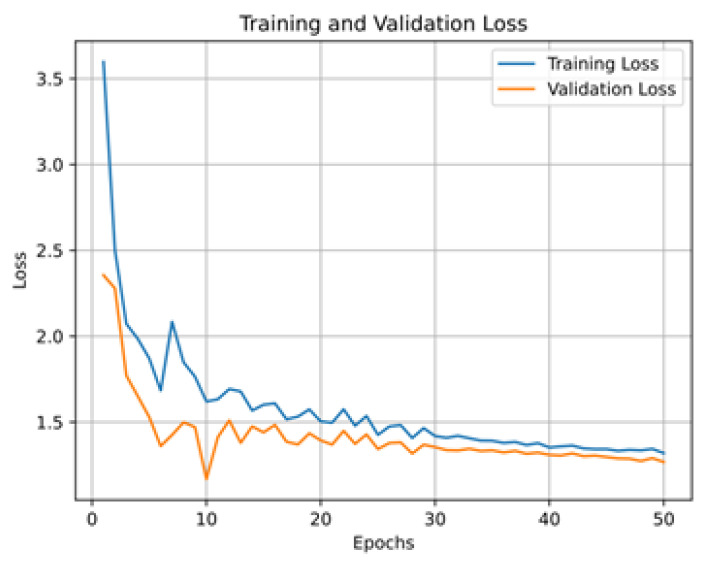
CNN sub-model validation loss.

Figure 5 presents the confusion matrix derived from the validation results of the CNN sub-model. This matrix offers a comprehensive visual representation of the CNN sub-model’s classification performance, highlighting the distribution of true positive, true negative, false positive, and false negative predictions across 10 different classes (i.e., the 10 labels for the 10 words listed in Table 2). Examining the confusion matrix provides crucial insights into the CNN sub-model’s ability to accurately classify instances within each class. As we can see, the true positives fell within the range of 82 to 97 when the CNN sub-model predicted the targeted word correctly. We can make an interesting observation from the confusion matrix: the CNN sub-model scored 89 and 92 TPs for labels 3 and 8, respectively. This is an indication that the CNN sub-model’s recognition performance was affected by viewing different angles. Furthermore, Figure 6 shows the loss evaluation for the training and validation of the CNN sub-model. The observed discrepancy in loss between the training and validation phases indicates that during the validation phase, the CNN sub-model became increasingly confident in interpreting hand gestures using hand landmarks as the number of epochs progressed.

### 5.3. LSTM Evaluation Metrics

**Figure 7:**
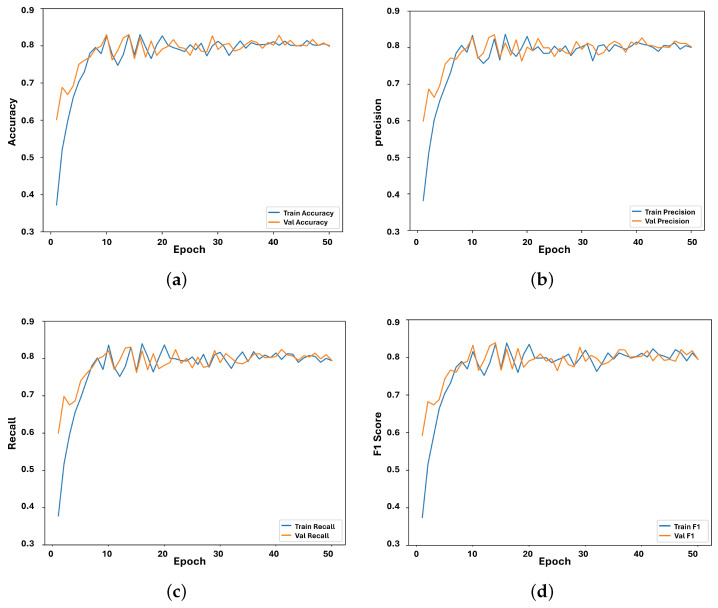
LSTM sub-model performance evaluation. (**a**) LSTM sub-model accuracy; (**b**) LSTM sub-model precision; (**c**) LSTM sub-model recall; (**d**) LSTM sub-model F1 score.

Figure 7 illustrates the results of the performance of the LSTM sub-model on the 10 words listed in Table 4. For this experiment, we trained the LSTM sub-model using 400 videos (i.e., for hand gesture sequences) and validated it using 100 videos. As we can see, the accuracy of the LSTM sub-model was 82.70% (see Figure 7a). The reason for this is the adequate amount of data for training, where the model could understand the hand gesture sequences. For this experiment, we also used 50 epochs to allow the LSTM sub-model to understand the targeted hand gesture sequences. We can also observe that the LSTM sub-model reached a precision of 84.20% (see Figure 7b) and recall and F1 scores of 82.50% and 82.70%, respectively, as shown in Figure 7c,d. The accuracy, precision, recall, and F1 scores consistently fell within the range of 78.50% to 82.70%. The consistency in performance across these metrics suggests well-balanced model behavior. The similarity in accuracy, precision, recall, and F1 score can be attributed to the balanced composition of the dataset, ensuring sufficient representation of each class. This balanced representation enabled fair evaluation of performance across all classes.

**Figure 8:**
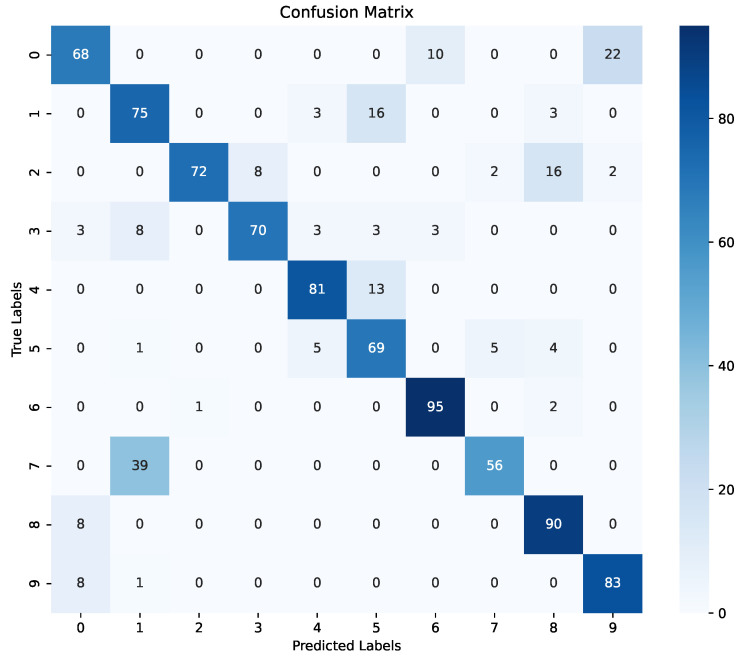
LSTM sub-model confusion matrix.

**Figure 9:**
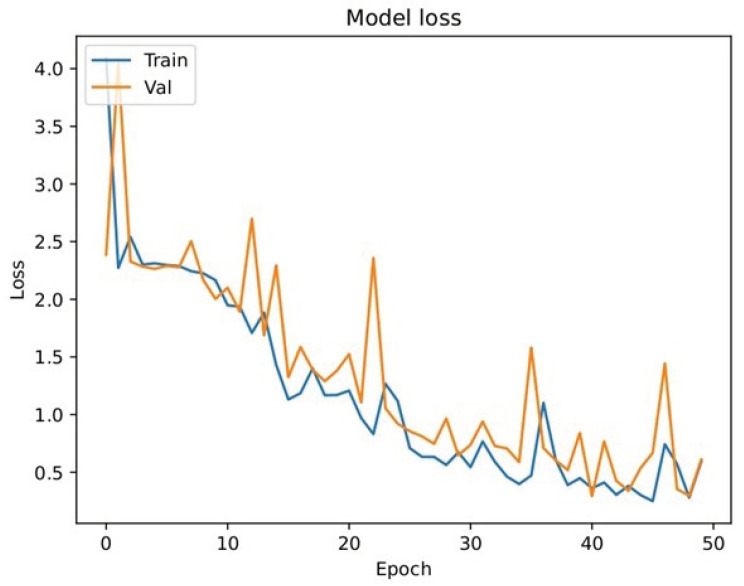
LSTM sub-model validation loss.

Figure 8 presents the confusion matrix derived from the validation results of the LSTM sub-model. This matrix offers a comprehensive visual representation of the LSTM sub-model’s classification performance, highlighting the distribution of true positive, true negative, false positive, and false negative predictions across 10 different classes (i.e., the 10 labels for the 10 words listed in Table 4). Examining the confusion matrix provides crucial insights into the LSTM sub-model’s ability to accurately classify instances within each class. As we can see, the true positives fell within the range of 56 to 95 when the LSTM sub-model correctly predicted the targeted word. Furthermore, Figure 9 shows the loss evaluation for the training and validation of the LSTM sub-model. We can see fluctuations in the loss outcomes between the training and validation phases, which can be attributed to the LSTM sub-model’s focus on extracting temporal features, such as motion signs, rather than spatial features, such as static signs, as observed in the CNN sub-model. Additionally, the loss results in the validation phase of the CNN sub-model (see Figure 6) exhibit superior performance compared to those of the LSTM sub-model, primarily due to this distinction in feature extraction.

## 6. Conclusions and Future Work

We examined the issue of the pronounced deficiency of sign language interpreters in the Kingdom of Saudi Arabia, highlighting the notable gap in the ratio of interpreters to individuals with hearing impairments compared to other regions like California, USA. This study revealed that this scarcity presents a significant obstacle in delivering services to the deaf community in public spaces. In this paper, we propose a hybrid model designed to capture both spatial and temporal elements of sign language. This hybrid model comprises a CNN classifier to extract spatial features from sign language data and an LSTM classifier to capture both the spatial and temporal characteristics essential for sequential data processing. By automating ArSL translation in real time between sign language and spoken or written language, this hybrid model aims to address the interpreter shortage.

To demonstrate the viability of our proposed hybrid model, we created a dataset of 20 different words, comprising 4000 images for 10 static gesture words and 500 videos for 10 dynamic gesture words. Our hybrid model showcased promising performance, with the CNN and LSTM classifiers achieving accuracy rates of 94.40% and 82.70%, respectively. One implication of our research is the superior performance of the CNN compared to LSTM, attributed to LSTM’s emphasis on extracting temporal features. At the same time, the CNN focuses on spatial features, such as static signs. In future work, we aim to expand the number of words in the datasets for both models in terms of both images and videos and use different viewing angles to enhance the performance of the sub-models utilized in our hybrid model. This research work holds the potential to enhance the availability of translation services, catering to the needs of the deaf community, thereby fostering effective communication, enhancing accessibility, and promoting solidarity with this significant segment of society.

## Figures and Tables

**Table 1:** List of configuration parameters and values.

Parameter	Value
Cloud service provider	Google Cloud
Instance type	Ubuntu 20.04
Operating system	CentOS Linux
CPU	Intel Xeon Platinum 8481C Processor
RAM	16 GB
Disk size	32 TB

**Table 2:** CNN data labeling.

Label	Name	Gesture
0	Hello/مرحبا	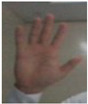
1	No/لا	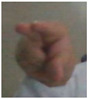
2	Agree/أتفق	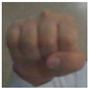
3	Awesome/مذهل	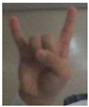
4	Good/جيد	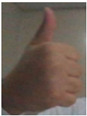
5	You/أنت	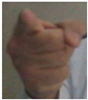
6	Bad/سيئ	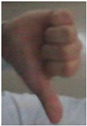
7	Question/سؤال	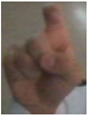
8	Not sure/لست متأكد	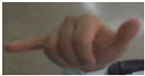
9	Excellent/ممتاز	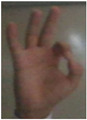

**Table 3:** CNN sub-model hyperparameter and loss function settings.

Hyperparameter	Value
Learning Rate	0.001
Batch Size	32
Number of Epochs	50
Optimizer	Adam
Loss function	Cross-Entropy
Dropout Rate	0.5
Data Augmentation	No

**Table 4:** LSTM data labeling.

Label	Name	Gesture
0	Peace be upon you/السلام عليكم	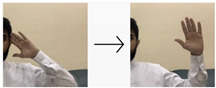
1	My name/أسمي	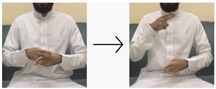
2	I want/أريد	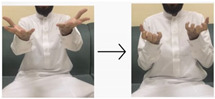
3	An exercise/تمرين	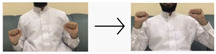
4	Hungry/جائع	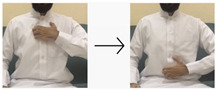
5	Work/عمل	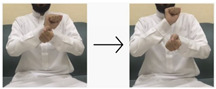
6	Book/كتاب	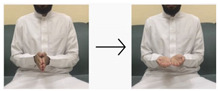
7	Water/ماء	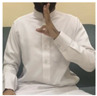
8	Teacher/معلم	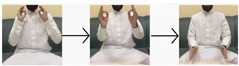
9	Teaches/يدرس	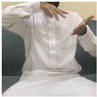

**Table 5:** LSTM sub-model’s layers and parameter setup.

Layer (Type)	Output Shape	Param. #
Lstm 1 (LSTM)	(None, 30, 64)	442,112
Lstm 2 (LSTM)	(None, 30, 128)	98,816
Lstm 3 (LSTM)	(None, 64)	49,408
Dense 1(Dense)	(None, 64)	4160
Dense 2 (Dense)	(None, 32)	2080
Dense 3 (Dense)	(None, 10)	330
Total params:		596,906

## Data Availability

The data is available on the following link (https://github.com/AhmedIbrahim110/Dataset).
